# Quality of independent review board/ethics committee oversight in clinical trials in India

**DOI:** 10.4103/2229-3485.80364

**Published:** 2011

**Authors:** Anvita Pandiya

**Affiliations:** *Clinical Research - Quality, Eli Lilly and Company (India) Pvt. Ltd., Plot No. 92, Sector 32, Gurgaon – 122 001, Haryana, India*

## ETHICS COMMITTEE FUNCTIONING IN INDIA: RESPONSIBILITIES AND CONCERNS

Independent Review Boards (IRBs) or Ethics Committees (ECs) have been entrusted with the responsibility to safeguard the rights, safety, and well-being of all trial subjects.[1] Constraints like lack of space, infrastructure, time, funds and administrative support result in most EC activities being restricted to an initial review and approval of study protocol,[2] although they are responsible for ongoing review and monitoring of trial activities to ensure that patient’s rights, safety and well-being are protected.

Some of the global concerns regarding trials being conducted in emerging markets are as follows. Is the clinical research responsive to the health needs and priorities of the communities in which the research is conducted? Are trial results accurate and valid, and can they be extrapolated to other settings? Is the financial compensation and health benefits provided to research participants an undue inducement? A major concern is the ethical oversight of research involving human subjects to ensure that these concerns are addressed.[3]

IRBs are the one entity that is still neglected with inadequate training, limited resources and no legal teeth. While personnel from sponsors are well trained in Good Clinical Practices (GCP) and sponsors also ensure training of investigators and site personnel, there is no formal training on bioethics mandated for EC members. The fear of a perceived nexus between sponsor/CRO and EC prevents sponsor/CRO sponsored training for EC members. In India, being an EC member is hardly ever a full-time job; so, resources for training are also limited. Due to this lack of training in bioethics, EC members do not have a clear understanding of complex ethical issues like reduced autonomy, cultural specificities in obtaining informed consent, vulnerable population, therapeutic misconception, conflict of interest, use of placebo, distributive justice, management of and compensation for study-related injury, and post trial access. Hence, ECs rarely go beyond a scientific review.[2,4]

Often, the head of the institution is a vocal member of the EC even if not in the capacity of the Chairperson (as per our regulations, the Chair should not be affiliated to the institution).[1,5,6] Besides, EC members do not declare financial or other relationships with industry. Hence, there is potential for bias and conflict of interest.

Though ethical guidelines have been established for a long time, many ECs are still grappling with basic issues like inadequate or no standard operating procedures (SOPs), constitution of EC not compliant with Schedule Y, irregular schedule of EC meetings, poor record keeping and archival, no processes in place for expedited review.[7]

A survey by ICMR was conducted in 2003, where a 20-point questionnaire was circulated to 1200 institutions of which only 223 responded. It concluded among other points that many research institutions in India either do not have an EC or there is inadequate representation in it by persons other than those of the medical fraternity.[7]

A study on profile of EC members concluded that EC members were generally senior in age, highly educated and well experienced in research, but representation of nonscientific members needed to be increased. Even with an appropriate EC constitution, the members had sub-optimal understanding of ethical issues and ethical principles.[8] However, it has been noticed that nonscientific members in most ECs do not participate actively in the deliberations of scientific and ethical merit of a study protocol. Probably, the very fact that there are senior, highly educated members from the medical fraternity and the lack of bioethics understanding inhibit the nonscientific members from voicing their opinion.

There is an increased scrutiny of clinical research quality from regulators, media and whistleblowers. Despite major strides taken in most aspects, including clear ethical guidelines, awareness and training of investigators and stringent monitoring by sponsors, adverse coverage by the media continues to plague the clinical research scenario in India.

Clinical research work in India has increased tremendously. Yet, regulatory reform and ethical practices have not kept pace. DCGI has announced plans of inspections of only sites, but not ECs. However, registration of ECs is in plan.

In the developed world, a critical observation during an auditor regulatory inspection could mean loss of reputation, being blacklisted from clinical research and even no marketing authorization for the drug.

In the last quarter of year 2010, the FDA has issued warning letters to two IRBs.[9]

The *first* was for issues such as the following.

1. Failure to ensure that the IRB is composed of at least five members, at least one IRB member’s primary concerns are in nonscientific areas, and no IRB member participates in the initial or continuing review of any projects in which the member has a conflict of interest. Failure to have adequate written procedures governing the functions and operations of the IRB.

The *second* enumerated the following.

2. Failure to require that information given to subjects as part of informed consent is in accordance with 21 CFR 50.25. Failure to follow written procedures for conducting its initial and continuing review of research. Failure to include at least one member whose primary concerns are in a nonscientific area when reviewing proposed research at convened meetings. 4) Failure to prepare and maintain adequate documentation of IRB activities, including minutes of IRB meetings.

Previous issues noted include failure to conduct continuing review in a timely or appropriate fashion; conflict of interest of IRB members; inappropriate use of expedited review; failure to inform IRB members of expedited approvals; inadequate attendance at and documentation of IRB meetings; standard surgical informed consent documents lacking the required elements; inappropriate granting of exempt status for studies involving prospectively collected specimens, data, documents, or records; and inappropriate granting of waivers of consent without documentation of compliance with the required criteria for approval.[10]

## SUGGESTIONS TO IMPROVE EC FUNCTIONING

It is in the best interest of all stakeholders (sponsors/CROs, investigator, institution, ECs, regulators, patients) as well as public health to conduct clinical research with quality. This will give greater confidence in the data generated. More importantly, better drugs and new treatment modalities would reach the patient faster.

Writers have been unanimous in stating that further investments and training of IRBs is a must for capacity building and ensuring that India continues to participate in global clinical trials.[3,5]

Other approaches to improve the conduct of clinical trials in India are also needed. Researchers and medical students should have clinical research methodology and bioethics as a part of their training. Patients’ groups need to be trained so that they can participate in setting the research agenda and in selecting outcomes relevant to their needs. ECs need to involve patient representatives in their decisions to oversee the consent process and protect patients’ rights and welfare. Research needs to have accountability to the public, which is currently lacking.[11]

The basics of any quality program is to develop processes to ensure quality, define controls to prevent errors, measure effectiveness, identify problems, include risks and develop interventions to correct and prevent occurrence of the problems/potential problems. It is difficult to establish parameters to assess the quality of ethical review, namely, protecting patient rights and safety, more so in India where ethics is still evolving.

Without a measure of ethical quality; institutions, institutional review boards, regulators, and the public have no way of knowing if the intent of regulations is being realized. There is also no way to determine ways to improve and achieve the goal of ethical oversight.[12]

A study conducted to identify the factors that influence commitment to the role and responsibilities of being on an IRB suggested that it may be related to adequate compensation for the time spent on IRB activities, serving on an IRB whose chair fosters a supportive group dynamic, IRB size, length of tenure, efficiency of protocol review, receiving training on the ethical and regulatory requirements for research with humans, and attendance of principal investigators at convened meetings. A certified IRB professional exam would go a long way in ensuring understanding of bioethics.[13]

The field of patient safety has adopted the systems approach for quality developed by the aviation industry to reduce system-based errors in the delivery of health care. There is no reason why ECs would not benefit from using something similar to protect patient safety and rights in the ethics domain. A checklist of the ethical issues that are inherent to clinical research and need to be assessed could be developed. A mere checking exercise would prompt the EC members to ensure that all ethical issues are given due thought. Checklists could go further to assess the degree of ethical concern by marking these on a scale of 1–5, with 1 meaning no ethical concerns and 5 meaning serious ethical concerns. Some of the ethical issues to be assessed are equitable selection of subjects, scientific merit of study, favorable risk benefit ratio, quality of information provided to subjects, and the informed consent process, factors likely to affect the voluntariness of potential subjects’ decisions about participation, and approaches to including subjects with limited or questionable decision-making capacity, fair subject selection, independent review, and respect for enrolled persons.[12]

The importance of bioethics and the role that ECs play is evident from the international focus on the same. Dr. Amy Gutmann, Chair of the Presidential Commission for the Study of Bioethical Issues, recently announced the formation of an International Research Panel to consider the standards for protecting human subjects in scientific studies. The announcement comes in direct response to a request from President Obama who asked the commission to report on the effectiveness of current US rules and international standards for the protection of human subjects in scientific studies supported by the Federal Government and to assure him that the current rules for research participants protect people from harm or unethical treatment, domestically as well as internationally. The International Research Panel includes experts on medical ethics, science and clinical research, who bring wide experience from academia, government, and industry. They hail from many countries, including Argentina, Brazil, China, Egypt, Guatemala, India, Russia, Uganda, Belgium, and the United States.[14]

Accreditation of IRBs is still some time away, but individual ECs could raise the bar of ethical review by adopting systematic means to measure how far the ethical goals were met. ECs should proactively ensure that their members receive ongoing training in bioethics.

Audits and inspections of EC/IRBs would focus attention on the deficiencies of their functioning and help improve quality. Sponsors could help the cause by reviewing EC SOPs and insisting on them following local regulations and guidelines before selecting the site that the EC is responsible to provide oversight for. It is the responsibility of all the stakeholders, especially the IRBs, to function effectively and restore public faith.
